# Use of the Anterolateral Thigh in Cranio-Orbitofacial Reconstruction

**DOI:** 10.1155/2011/941742

**Published:** 2011-11-10

**Authors:** William J. Parkes, Howard Krein, Ryan Heffelfinger, Joseph Curry

**Affiliations:** Department of Otolaryngology-Head and Neck Surgery, Thomas Jefferson University Hospital, 925 Chestnut Street 6th Floor, Philadelphia, PA 19107, USA

## Abstract

*Objective*. To detail the clinical outcomes of a series of patients having undergone free flap reconstruction of the orbit and periorbita and highlight the anterolateral thigh (ALT) as a workhorse for addressing defects in this region. *Methods*. A review of 47 patients who underwent free flap reconstruction for orbital or periorbital defects between September 2006 and May 2011 was performed. Data reviewed included demographics, defect characteristics, free flap used, additional reconstructive techniques employed, length of stay, complications, and follow-up. The ALT subset of the case series was the focus of the data reviewed for this paper. Selected cases were described to highlight some of the advantages of employing the ALT for cranio-orbitofacial reconstruction. *Results*. 51 free flaps in 47 patients were reviewed. 38 cases required orbital exenteration. The ALT was used in 33 patients. Complications included 1 hematoma, 2 wound infections, 3 CSF leaks, and 3 flap failures. *Conclusions*. Free tissue transfer allows for the safe and effective reconstruction of complex defects of the orbit and periorbital structures. Reconstructive choice is dependent upon the extent of soft tissue loss, midfacial bone loss, and skullbase involvement. The ALT provides a versatile option to reconstruct the many cranio-orbitofacial defects encountered.

## 1. Introduction

Extirpative surgery involving the orbit and periorbita often results in a complex defect involving multiple midfacial subsites. Depending upon the extent of resection, the reconstructive surgeon may need to address any combination of the following: periorbital skin (forehead, nose, and midface), orbital soft tissue, supra orbital or midface bone, paranasal sinuses, and anterior skull base. A multitude of reconstructive options are available including skin grafting and regional flaps, such as the temporalis muscle or temporoparietal fascia. Yet, in some cases the volume of soft tissue loss or complexity of the defect may require the versatility afforded by free tissue transfer for optimal reconstruction. A variety of free flaps may be employed to this end, including the anterolateral thigh (ALT), radial forearm (RF), fibula, and latissimus dorsi. At our institution, we favor the use of the ALT. The ALT functions to allow a variety of soft tissue configurations very useful to periorbital reconstruction and is the subject of this work.

First described by Song et al. [1] in 1984, the ALT has become a workhorse in head and neck reconstruction, allowing for a two-team approach and offering versatility, ample pedicle length, and low donor site morbidity. The ALT can be harvested as a fasciocutaneous, myocutaneous, subcutaneous, or adipofascial flap. The perforators supplying the flap typically arise from the descending branch of the lateral circumflex femoral artery (LCFA), but anatomic variation is not infrequently encountered and must be recognized. Yu [2] described and defined 3 distinct types of ALT perforator systems.Type I perforators arose from the descending branch of the LCFA and accounted for 90% of perforators identified in their series. Type II perforators arose from the transverse branch of the LCFA and were encountered roughly 4% of the time. Lastly, Type III perforators arose directly from the profunda femoris artery and were too small for microvascular anastomosis. The perforators can be either septocutaneous or musculocutaneous. In their large series, Wei et al. [3] reported that 87.1 % of 504 fasciocutaneous or cutaneous ALT flaps were perfused by musculocutaneous perforators, while 12.9 % were perfused by septocutaneous perforators. The ALT has an average pedicle length of 7-8 cm with a range in length up to 16 cm and a vessel diameter of larger than 2 mm [4]. Donor defects with a medial to lateral diameter less than 8-9 cm are typically closed primarily [5].

 With respect to the orbit, the ALT provides sufficient soft tissue and skin to fill the dead space within an exenteration cavity and to cover any implants or plates required for adjacent midface reconstruction. Multiple perforators and the technique of de-epithelialization allow for the creation of separate islands within one flap so that each portion (i.e., skin, exenteration cavity, and maxillary sinus) of the overall defect can be addressed separately. Most importantly, the ALT provides effective coverage for anterior skull base defects. Both the vastus lateralis fascia and the adjacent tensor fascia lata can be harvested and used to reconstitute the dural layer when necessary [6, 7]. 

This retrospective review details the clinical outcomes of a series of patients having undergone free flap reconstruction of the periorbita and highlights the ALT as a workhorse for addressing defects in this region. 

## 2. Patients and Methods

A review of 47 patients who underwent 51 free flaps to reconstruct orbital or periorbital defects between September 2006 and May 2011 was performed at our tertiary care facility. Thirty-five patients were male and 12 were female. The average age at the time of surgery was 65. The majority of the resections were completed for invasive cutaneous malignancies. The spectrum of pathology encountered in the series is outlined in Table 1. 

All defects reconstructed in the series involved skin and soft tissue of the orbit and periorbita (defined as paranasal sinuses, midface bone, and/or anterior cranial base). Thirty-eight out of 47 (81%) extirpations required complete orbital exenteration, which was conducted by ophthalmology, while the free flap harvest was underway. In 22 cases, resection exposed the dura of the anterior cranial base, and in half of those instances, neurosurgery placed a lumbar drain. Additional reconstructive techniques employed throughout the series included 12 local flaps, 6 recon plates, 3 Medpor implants, 1 bone graft, and 2 skin grafts.

Data reviewed included demographics, type of free flap used, recipient vessels, length of stay, length of follow-up and complications. Complications were defined as wound infection, hematoma, CSF leak, or flap loss. Data collection and analysis were performed with Institutional Review Board approval. For this paper, attention was paid to the ALT subset of the case series during data review.

Each ALT harvest was planned in the standard fashion by centering an elliptical incision at the midpoint of a line between the anterior superior iliac spine and the superolateral patella. A Doppler stethoscope was then used to identify cutaneous perforators, which were marked on the skin surface. Harvesting began with an incision along the medial half of the ellipse and dissection was carried down through the subcutaneous fat until the investing fascia of the rectus femoris muscle (RFM) was identified. Dissection then proceeded toward the intermuscular septum between the RFM and the vastus lateralis muscle (VLM) by retracting the investing fascia laterally and the RFM fibers medially. In this plane, the descending and transverse branches of the lateral circumflex femoral artery were identified and preserved. The perforators, whether septocutaneous or, more commonly, musculocutaneous, were then carefully identified and traced to the main pedicle. Musculocutaneous perforators were followed with a Harmonic scalpel via an unroofing technique. All 3 types of perforator systems as defined by Yu et al. were encountered during out experience. Once the course of the perforators was confirmed, the lateral skin incision was made and taken down to the VLM in the subcutaneous plane. A fasciocutaneous flap was then raised, leaving most of the VLM behind. Lastly, the pedicle was followed proximally and clipped just distal to the take-off of the main supply to the RFM. 

## 3. Results

Free flaps used in our series are as follows: 33 (65%) ALT flaps, 13 (25%) RF flaps, 4 (8%) latissimus dorsi flaps, and 1 (2%) rectus abdominus flap. Forty-seven of the arterial pedicles were anastomosed end to end with branches of the external carotid, and 4 were sewn end to side into the external carotid itself. The most common recipient vessels were the superficial temporal artery (20 cases) and the facial artery (21 cases). Other arteries used included the occipital, ascending pharyngeal, and superior thyroid. In the ALT subset, the superficial temporal artery (13 cases) and the facial artery (13 cases) were again the most commonly employed recipient vessels. Arterial grafts were not required in any of the cases. Recipient veins included the internal jugular, external jugular, superficial temporal, facial, and retromandibular. One saphenous vein graft was required. Average length of stay and length of follow-up (based on the date of the most recent clinical encounter found on either the inpatient or ambulatory electronic medical record) were 10 days and 14 months, respectively. In the ALT subset, the average length of stay and length of follow-up were 11 days and 14 months, respectively.

In terms of minor complications, there were 3 instances of wound infection and 3 hematomas over the entire case series. All 3 wound infections were treated with surgical drainage and debridement, and all 3 hematomas were treated with surgical evacuation. Two of the wound infections and one of the hematomas occurred in the ALT subset to give an incidence of 6% and 3% for infection and hematoma in that patient population. 

CSF leak was documented in 3 out of 18 cases involving anterior cranial base resection followed by ALT flap reconstruction. One leak was low flow and was managed conservatively with the placement of a lumbar drain postoperatively. The remaining two cases were high flow and required a return trip to the operating room with neurosurgery for a formal skull base repair using a vascularized nasal septal flap in one instance and a fat graft combined with a pericranial flap in the other.

Lastly, there were 3 instances of ALT flap loss, which translates to a 91% success rate in the ALT subset of the series. Of the 3 losses, 2 were recognized and managed intra-operatively while the other was found to have necrosed at the first follow-up visit on postoperative day 18. The first intra-operative loss was reconstructed with a RF flap during the same procedure, while the second was reconstructed with a staged latissimus dorsi flap performed the following day. 

## 4. Case Studies

### 4.1. Case 1

**Figure 1:**
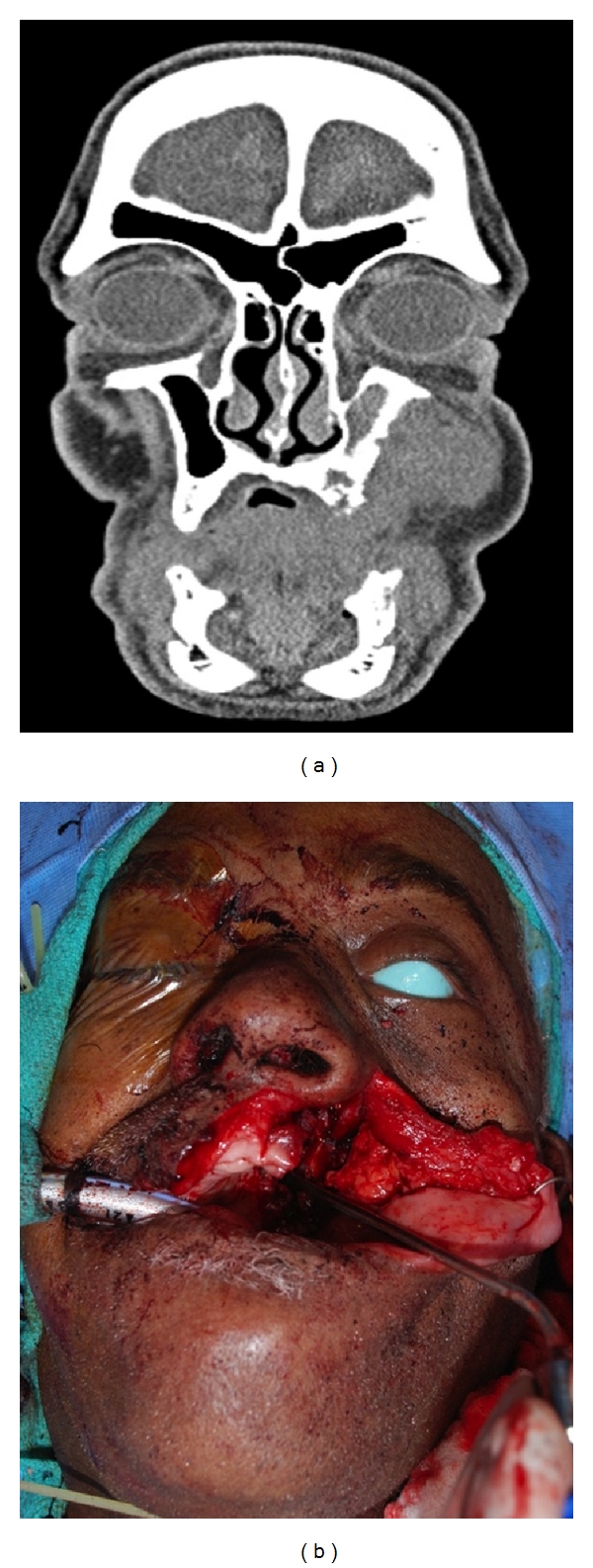
Preoperative coronal CT scan demonstrating invasive midface lesion and resultant defect after extirpation.

**Figure 2:**
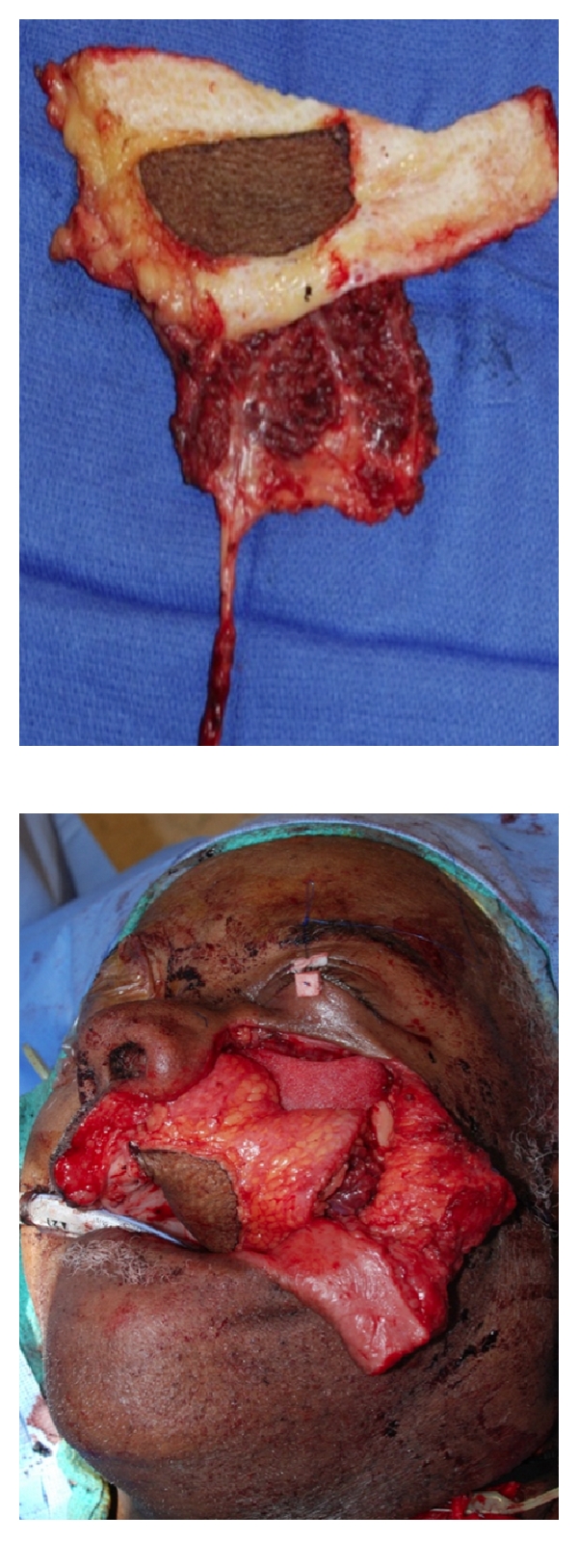
Flap design highlighting the de-epithelialization technique for multisubsite reconstruction and subsequent inset with Medpor orbital rim implant visible.

*The ALT provides sufficient soft tissue and skin to fill dead space and cover any implants or plates required for adjacent malar complex and orbital reconstruction. Additionally, the ALT can be split into multiple paddles for insetting separate subsites via the deepithelialization technique*. A 79-year-old male presented with an advanced squamous cell carcinoma of the left maxillary sinus with invasion into the palate, infratemporal fossa, pterygomaxillary space, and infraorbital rim. Extirpation preserved the globe but created a complex defect involving the entire maxilla, the palate, a portion of the pharyngeal wall, the infraorbital rim, and the orbital floor (Figure 1). A large segment of the flap was de-epithelialized and used to replace the missing maxillary volume, to line the lateral nasal sidewall, and to provide coverage for a Medpor orbital rim implant (Figure 2). The midportion of the cutaneous pedicle was left intact to recreate the oral lining and was inset to the palatal and buccal mucosa. The vascular pedicle was tunneled subcutaneously and anastomosed to the facial vessels in the left neck. Vein grafts were not necessary. A Merocel pack was placed in the left nose to prevent the flap from collapsing into the nasal cavity. A left tarsorrhaphy via a Frost stitch was also performed to prevent traction on the lower lid.

### 4.2. Case 2

*If a significant anterior skull base defect is present, a portion of the ALT fascia can be utilized to reconstitute the dura*. A 52-year-old male presented with a recurrent malignant meningioma of the left orbit and anterior cranial fossa. A left-sided orbital exenteration and bifrontal craniotomy for anterior skull base resection were performed, leaving a large defect with a significant amount of exposed dura. A fasciocutaneous ALT was harvested, and the entire flap was de-epithelialized. Fascia from the RFM and VLM was used to recreate the floor of the entire anterior cranial fossa, and the de-epithelialized dermis was inset facing inferiorly to line the remaining sinonasal cavity and recreate the left lateral nasal sidewall. Residual fat was used to fill the dead space of the exenteration cavity, which was then closed over using the lid apparatus. The pedicle was tunneled out through the malar soft tissue to reach the distal facial artery, as the superficial temporal artery was not available on either side secondary to previous bifrontal craniotomy. During the harvest, it was clear that the dominant vessel to the skin arose from the transverse branch of the LCFA rather than the descending branch. Despite the fact that the flap was raised off of this branch, there was adequate length to reach the facial artery and vein. Neurosurgery re-secured the frontal and supra orbital rim bone plate and placed a lumbar drain. At the conclusion of the procedure, a Merocel pack was inserted into the left nasal cavity and a nasal trumpet was placed in the right nasal cavity to limit air tracking along the reconstructed anterior cranial base.

### 4.3. Case 3

**Figure 3:**
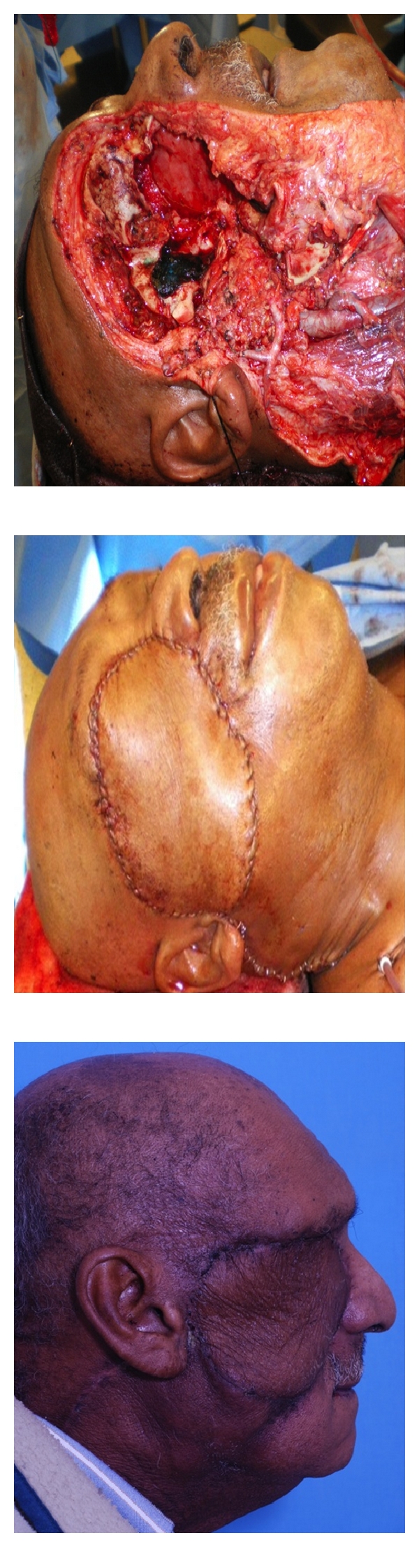
Large defect after orbital exenteration exposing the anterior cranial base, immediate postop result in operating room, and result at 6 weeks postop.

*Long-term results of cranio-orbitofacial reconstruction with the ALT flap are aesthetically sound.* A 72-year-old male presented with a history of a progressively enlarging mass of the right-sided temporal and orbital region. Imaging revealed invasion into the right orbit, maxillary sinus and middle fossa cranial base. Extirpation involved an orbital exenteration, total maxillectomy, craniotomy, resection of facial musculature in the midface and sacrifice of the facial nerve. Prior to inset, the lateral and superior orbital rims were reconstituted with titanium mesh, and the right oral commissure was then suspended from this plate with a 2-0 Prolene suture. Of note, the ALT harvested was supplied by 2 distinct vascular pedicles (1 from the descending branch of the LCFA and 1 from the transverse branch of the LCFA). Because of this, 5 anastomoses were required (2 arterial and 3 venous). The defect, immediate postoperative result and long-term postoperative result, is displayed (Figure 3).

## 5. Comment

A wide variety of reconstructive options are available for defects of the periorbital region. For larger more complex defects, free tissue transfer may have several advantages over most other modalities, especially with respect to the volume of tissue readily available for use, the variety of tissue available, and the ability to incorporate and provide coverage for plates and implants. A variety of flaps may be considered, including the ALT, RF, fibula, subscapular system flaps, or rectus abdominus. The ALT in particular offers several appealing qualities that make it a highly useful option for periorbital reconstruction.

With regards to the harvest, flap raising is quick and easily performed with a 2-team approach, which is generally not as feasible for subscapular system flaps. Unlike the RF and fibula flaps, no tourniquet is necessary. In addition, the wound for the thigh is generally closed primarily in most patients, thereby avoiding the need for a skin graft, which is required with the fibula or forearm flaps. This also avoids the need for a splint, bolster, wound VAC, or cast, thus simplifying postoperative wound care. 

Many patients tolerate the harvest quite well and are ambulatory as soon as they are mobilized, which is generally postoperative day 2. To minimize any functional deficit in the quadriceps muscle group, we prefer to harvest a predominantly fasciocutaneous flap and limit the inclusion of vastus musculature unless required to preserve perforators. Using objective kinetic analyses, Kuo et al. have demonstrated a statistically significant difference in functional morbidity favoring a vascularized fascia ALT flap over a myocutaneous ALT flap [8]. Furthermore, excessive muscle harvest is unlikely to offer a significant increase in flap bulk given the inevitable atrophy that will ensue secondary to denervation. When motor nerves are sacrificed because of aberrant perforator anatomy, we prefer to reconstruct them with primary coaptation or nerve graft. The most commonly seen postoperative complication is seroma of the thigh, typically encountered several weeks after surgery once the patient has become increasingly ambulatory. This is a minor morbidity causing minimal patient discomfort that is easily treated with needle drainage in the office.

**Figure 4:**
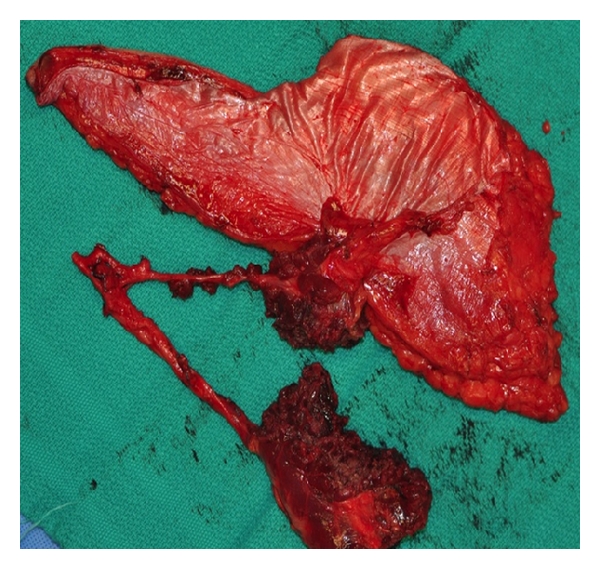
ALT flap immediately after harvest showing the use of multiple perforators to create separate islands.

 While ease of flap harvest and low donor site morbidity are two of the workhorse attributes of the ALT, tissue versatility is the primary reason for which it is well suited for periorbital reconstruction. Harvest frequently yields a flap of >200 cm^2^, with harvests as large as 20 cm (in width) by 26 cm (in length) reported in the literature [9]. With this much tissue available, the flap can accomplish a great variety of goals. By de-epithelializing the majority of the flap, leaving only enough skin to recreate the surface of the orbit, there is ample bulk to fill the dead space of an exenteration cavity, even without the harvest of much muscle. Although simpler techniques, such as skin grafting, can line an exenteration cavity, the vascularized free flap reduces the risk of bony exposure and the pursuant osteomyelitis that can occur. Additionally, the end result is low maintenance, allowing the patient to avoid chronic care and frequent decrusting often necessary with more limited reconstruction. To preserve orbital concavity in anticipation of a prosthesis, the ALT can also be carefully thinned either primarily or secondarily [10, 11]. As demonstrated in the described case, the ALT provides more than adequate soft tissue to line the entire floor of the anterior cranial fossa using the muscular fascia or adjacent tensor fascia lata. Lastly, periorbital defects are often large enough to involve noncontiguous subsites, such as an exenteration cavity and the oral cavity. The ALT can be divided into separate islands based on multiple perforators (Figure 4) or de-epithelialized to create separate segments on one large flap. De-epithelialization of the underlying surfaces is the easier of the two techniques and remains feasible when only a single perforator supplies the entire flap.

 Shortcomings of the ALT for periorbital reconstruction must be noted and most obviously include the lack of bone as a tissue option. Though chimeric ALT flaps that include a small piece of the iliac crest or the femur have been described [12, 13], these techniques are not widely practiced, and we have not elected to use them. Rather, we have favored the combined use of implants for orbital and midfacial bony reconstruction when necessary. When substantial bone loss in the hard palate is expected in patients for whom dental rehabilitation is a viable option, the fibula free flap would be the preferred option. However, in cases where extensive soft tissue is needed and functional bone loss is less likely (i.e., when there is no resection of the midfacial buttresses), then frequently, the ALT is a viable option. And when periorbital extirpation involves the hard palate in a patient for whom we suspect dental implants are unlikely, then an ALT can be augmented with a denture to provide functional chewing.

 Variable anatomy of the ALT donor site is also frequently pointed out as a downside, but the anatomy has been well described by Wong and Wei [14]. In their recent review article, they outline a “free-style” approach that essentially safeguards against the vascular aberrations. In most cases, a branch of the descending LCFA supplies one or several perforators to the skin, but this skin may also be supplied not infrequently by the transverse branch of the common femoral artery or an oblique branch. And at times, a single large perforator may supply an extremely large segment of the skin. Preoperative Doppler stethoscope identification of the perforators, adaptation of the flap skin harvest to these perforators, and careful dissection of the perforators with clear visualization of their entire course to the main pedicle allows for a reliable harvest despite alterations in anatomy.

Given the comorbidities of the patient population and the pathology encountered in extensive cranio-orbitofacial extirpations, complications and flap failures will certainly occur. With respect to the 3 CSF leaks encountered in our series, each case was for recurrent meningioma and required intradural resection. All were fixed successfully on the same admission with 2 of them requiring operative repair as mentioned above. In both cases that required a return trip to the operating room, the primary dural closure after the initial resection was accomplished with a DuraGen graft and not by vascularized fascia from the ALT flap. It is recommended that, when possible, primary dural closure with vascularized tissue should be accomplished. 

 In terms of the 3 flap failures, 2 were recognized intraoperatively. The third failure occurred after discharge in the setting of a complex infection. Interestingly, in both cases of intra-operative loss, ALT harvest was aborted in the opposite thigh because of aberrant anatomy. In both instances, the flap was ultimately deemed unsalvageable because of a no reflow phenomenon. Given these findings, an incomplete understanding of aberrant vascular anatomy likely led to immediate flap failure in these 2 patients. By employing the “free-style” approach to harvest referenced above, unique perforator systems can be better appreciated and the risk of acute loss can be reduced. 

To conclude, the workhorse attributes and tissue versatility of the ALT free flap provide the reconstructive surgeon with a safe and effective means of reconstructing complex cranio-orbitofacial defects.

## Figures and Tables

**Table 1:** Pathology in 47 patients undergoing cranio-orbitofacial extirpation with free flap reconstruction.

Pathology	All patients (*n* = 47)
Squamous cell carcinoma	
cutaneous	12 (26%)
sinonasal	5 (11%)

Basal cell carcinoma	6 (13%)

Melanoma	
cutaneous	3 (6%)
mucosal	1 (2%)

Meningioma	4 (9%)

Sinonasal adenocarcinoma	3 (6%)

Lacrimal sac carcinoma	2 (4%)

Adenoid cystic carcinoma, adenomucinous carcinoma, basosquamous carcinoma, esthesioneuroblastoma, frontal bone osteomyelitis, hemangiopericytoma, lacrimal gland carcinoma, mucor, peripheral nerve sheath tumor, schwannoma, SNUC	11 (1 each)

## References

[1] Song YG, Chen GZ, Song YL (1984). The free thigh flap: a new free flap concept based on the septocutaneous artery. *British Journal of Plastic Surgery*.

[2] Yu P (2004). Characteristics of the anterolateral thigh flap in a western population and its application in head and neck reconstruction. *Head and Neck*.

[3] Wei FC, Jain V, Celik N, Chen HC, Chuang DCC, Lin CH (2002). Have we found an ideal soft-tissue flap? An experience with 672 anterolateral thigh flaps. *Plastic and Reconstructive Surgery*.

[4] Malhotra K, Lian TS, Chakradeo V (2008). Vascular anatomy of anterolateral thigh flap. *Laryngoscope*.

[5] Lueg EA (2004). The anterolateral thigh flap: radial forearm's “big brother” for extensive soft tissue head and neck defects. *Archives of Otolaryngology*.

[6] Weber SM, Kim JH, Wax MK (2007). Role of free tissue transfer in skull base reconstruction. *Otolaryngology*.

[7] Rodríguez-Vegas JM, Ángel PA, Manuela PR (2008). Refining the anterolateral thigh free flap in complex orbitomaxillary reconstructions. *Plastic and Reconstructive Surgery*.

[8] Kuo YR, Yeh MC, Shih HS (2009). Versatility of the anterolateral thigh flap with vascularized fascia lata for reconstruction of complex soft-tissue defects: clinical experience and functional assessment of the donor site. *Plastic and Reconstructive Surgery*.

[9] Yildirim S, Avci G, Aköz T (2003). Soft-tissue reconstruction using a free anterolateral thigh flap: experience with 28 patients. *Annals of Plastic Surgery*.

[10] Koshima I, Fukuda H, Yamamoto H (1993). Free anterolateral thigh flaps for reconstruction of head and neck defects. *Plastic and Reconstructive Surgery*.

[11] Kimura N, Satoh K, Hasumi T, Ostuka T (2001). Clinical application of the free thin anterolateral thigh flap in 31 consecutive patients. *Plastic and Reconstructive Surgery*.

[12] Strauch B, Yu H-L (2006). *Atlas of Microvascular Surgery: Anatomy and Operative Approaches*.

[13] Acartürk TO (2011). Femur-vastus intermedius-anterolateral thigh osteomyocutaneous composite chimeric free flap: a new free flap for the reconstruction of complex wounds. *Journal of Reconstructive Microsurgery*.

[14] Wong C-H, Wei F-C (2010). Anterolateral thigh flap. *Head and Neck*.

